# An Investigation into the Dynamic Recrystallization (DRX) Behavior and Processing Map of 33Cr23Ni8Mn3N Based on an Artificial Neural Network (ANN)

**DOI:** 10.3390/ma13061282

**Published:** 2020-03-12

**Authors:** Zhongman Cai, Hongchao Ji, Weichi Pei, Xuefeng Tang, Long Xin, Yonghao Lu, Wangda Li

**Affiliations:** 1 College of Mechanical Engineering, North China University of Science and Technology, 21 Bohai Road, Caofeidian Xincheng, Tangshan 063210, China; czmncst@163.com (Z.C.); pwc@ncst.edu.cn (W.P.); lwd13731542189@163.com (W.L.); 2 National Center for Materials Service Safety, University of Science and Technology Beijing, Beijing 100083, China; long_xin@ustb.edu.cn; 3 State Key Laboratory of Materials Processing and Die & Mould Technology, Huazhong University of Science and Technology, 1037 Luoyu Road, Wuhan 430074, China; xftang@hust.edu.cn

**Keywords:** artificial neural network, austenitic stainless steel, dynamic recrystallization, grain size, processing map, sensitivity analysis

## Abstract

Based on an 33Cr23Ni8Mn3N thermal simulation experiment, the application of an artificial neural network (ANN) in thermomechanical processing was studied. Based on the experimental data, a microstructure evolution model and constitutive equation of 33Cr23Ni8Mn3N heat-resistant steel were established. Stress, dynamic recrystallization (DRX) fraction, and DRX grain size were predicted. These models were evaluated by a variety of statistical indicators to determine that these models would work well if applied in predicting microstructure evolution and that they have high precision. Then, based on the weight of the ANN model, the sensitivity of the input parameters was analyzed to achieve an optimized ANN model. Based on the most widely used sensitivity analysis (SA) method (the Garson method), the input parameters were analyzed. The results show that the most important factor for the microstructure of 33Cr23Ni8Mn3N is the strain rate ($\dot{\varepsilon }$). For the control of the microstructure, the control of the $\dot{\varepsilon }$ is preferred. ANN was applied to the development of processing map. The feasibility of the ANN processing map on austenitic heat-resistant steel was verified by experiments. The results show that the ANN processing map is basically consistent with processing map based on experimental data. The trained ANN model was implanted into finite element simulation software and tested. The test results show that the ANN model can accurately expand the data volume to achieve high precision simulation results.

## 1. Introduction

With the advent of the Internet age, computer technology has continued to evolve, and the artificial neural network (ANN) was born. At present, the combination of ANN and various fields is a hot research topic. ANN models and different optimization techniques are associated with success for a wide range of engineering problems [1,2]. Among the many neural network types, the error back propagation ANN (BP-ANN) is the most mature ANN [3]. It is known for its mature technology and wide range of applications. At present, ANN has been applied in research on the constitutive equation [4,5,6,7] and the prediction of microstructure evolution [8,9] in the thermal deformation of metallic materials. These methods, establishing the constitutive model of metal materials by ANN, can reduce the limitations of traditional regression models, and this has been recognized by most domestic and foreign scholars [10,11,12,13]. However, there are few reports on the application of an ANN to a dynamic recrystallization (DRX) model. At present, the prediction of microstructure evolution mostly uses the typical kinetic model. For example, Quan et al. [14] studied the DRX behavior of an AlCu4SiMg alloy by the Johnson-Mehl-Avrami-Kolmogorov (JMAK) type equation. The feasibility of the JMAK equation in an AlCu4SiMg alloy was verified by finite element method (FEM) and experiments. Cai et al. [15] established the DRX kinetic model of an AZ41M magnesium alloy by the Avrami equation to provide predictive guidance for DRX behavior. At the same time, some scholars have improved the existing classical methods or proposed new methods. For example, Liu et al. [16] proposed a new DRX kinetic model, which is characterized by dividing DRX behavior into three stages, and confirming the feasibility of the new model through experimental verification. Wen et al. [17] proposed a new DRX kinetic model that is characterized by the ability to quantitatively analyze the effects of δ versus DRX behavior. Chen et al. [18] proposed a new method for establishing DRX kinetic equations. The difference to the classical DRX kinetic model is that only the middle DRX volume fraction (X_drx_) of the deformed part is required to establish a DRX kinetic model. All of the above attempts were to improve typical kinetic models or propose new typical kinetic models. There are few reports on the application of an ANN in DRX behavior, so this paper has made an attempt in this aspect. 

The main influencing factors for microstructures currently known are strain (ε), strain rate ($\dot{\varepsilon }$), and temperature (T). These factors have a close relationship to the quality of the formed parts. The control of the forming quality is the control of the deformation parameters. Although it is currently known that deformation parameters have a major impact on processing quality, their importance has not yet been quantitatively evaluated. Sensitivity analysis (SA) is able to quantify the importance of input parameters for output parameters. SA is widely used in various fields such as in maritime related fields [19,20], environmental pollution [21], and renewable energy [22]. Although SA has been widely used in various fields, there are few reports on the application of SA in thermomechanical processing. SA contributes to the optimization of a model and the control of the forming quality, which has great potential for applications in thermomechanical processing. At present, the main SA methods are Garson [23], Morris [24,25], and Sobol [26,27]. Based on the Garson method, this paper will analyze the sensitivity of deformation conditions and determine the importance of different parameters to provide theoretical guidance for 33Cr23Ni8Mn3N heat-resistant alloy steel.

In thermomechanical processing, processing parameters play a decisive role in the quality of the part. However, the determination of optimal process parameters has always been a problem. A large number of tests are carried out through trial and error, which wastes manpower and resources and is not efficient. The emergence of processing maps has changed this situation. A processing map is established based on the experimental data, and the optimal process parameter interval can be given for the specific material to ensure the product quality. However, processing maps also have limitations, which include the only single method of establishing processing maps as well as the problems of limited amount of data. In this regard, some scholars have tried to apply ANNs to processing maps. For example, Yu et al. [28] constructed the ANN processing map of TC21, which confirmed that the power dissipation map and the unstable region of the ANN processing map are basically consistent with the processing map based on experimental data. Moreover, the ANN processing map reflects a stronger information ability. Yu et al. [29] established the ANN processing map of a Ti40 titanium alloy and verified the corresponding regional microstructure. Quan et al. [30] applied an ANN processing map to the 7075 aluminum alloy to verify the applicability of the method to aluminum alloys. In addition, it has been verified by experiments that ANNs have great potential in thermomechanical processing. At present, the exploration of ANN processing maps is mainly focused on titanium alloys and aluminum alloys, and an exploration of austenitic heat-resistant steels has not been done. Therefore, a similar attempt has been made here to confirm the feasibility of an ANN processing map in austenitic steel.

In this paper, various applications of ANNs in thermomechanical processing are carried out. Based on the thermal simulation experimental data, a predictive model for the DRX behavior of a 33Cr23Ni8Mn3N heat-resistant alloy steel is established using an ANN. The accuracy of the ANN prediction model is verified by experiments. The importance of different experimental parameters for DRX behavior is determined by SA (Garson). The ANN processing map is applied to austenitic heat-resistant steel to verify its feasibility for austenitic heat-resistant steel. The ANN model is developed in finite element software, and experimental simulation is carried out to verify its feasibility in finite element simulation.

## 2. Experiments and Materials

**Figure 1:**
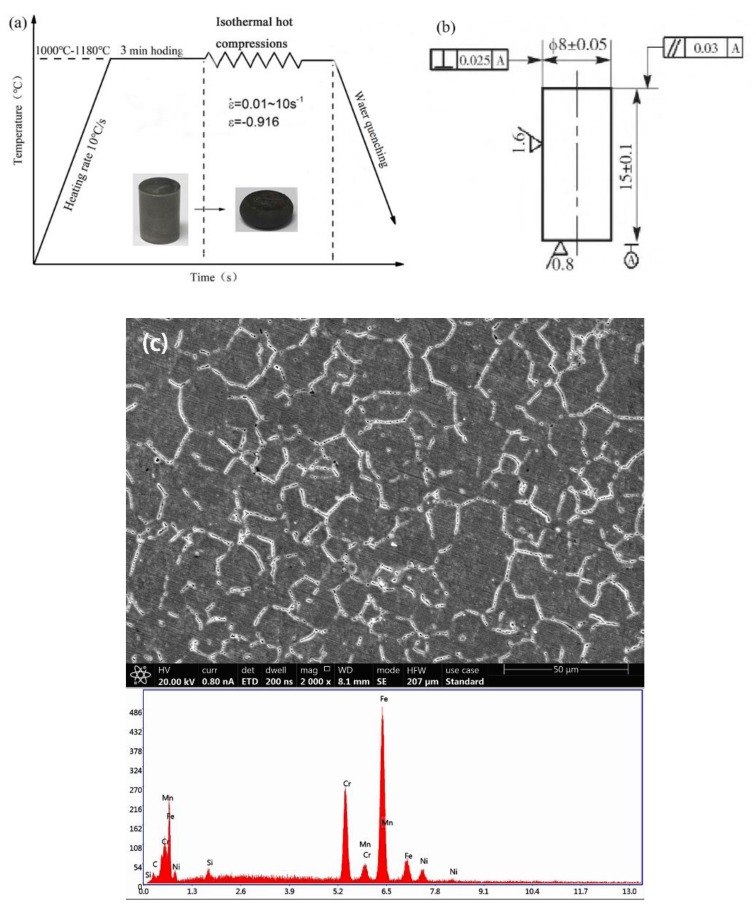
(**a**) Experimental scheme, (**b**) workpiece size, (**c**) and original metallographic structure of the 3Cr23Ni8Mn3N steel and energy spectrum diagram.

**Figure 2:**
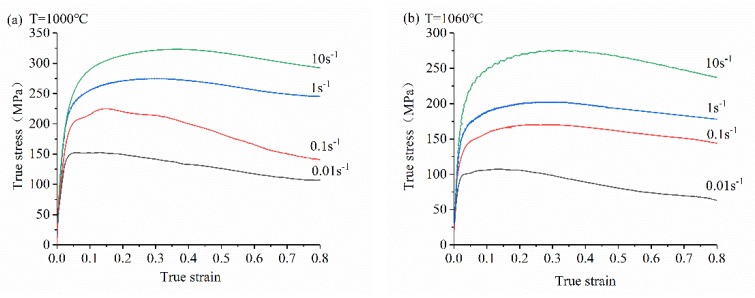

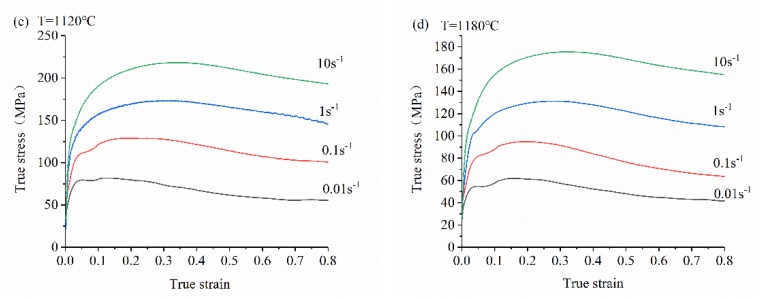
True stress-strain curves of 33Cr23Ni8Mn3N at different deformation conditions.

The material composition used in this experiment is as follows (wt %): C-0.38, Si-0.95, Mn-2.3, S ≤ 0.03, P ≤ 0.04, Cr-24, Ni-7.02, N-0.38, and Fe (balance). The experimental scheme, workpiece size, original metallographic structure, and energy spectrum diagram are shown in Figure 1. The workpiece was deformed under different conditions according to the experimental plan to obtain the required experimental data. The experimental process is: the test piece is heated to the experimental temperature (1000, 1060, 1120, and 1180 °C) at a heating rate of 10 °C/s, held for 3 min to homogenize the temperature. Hot compression tests were performed at different strain rates (0.01, 0.1, 1, and 10 s^−1^). The experiment was completed and water quenched immediately to ensure that the microstructure no longer changed [31]. Under experimental conditions, the stress-strain curve of 33Cr23Ni8Mn3N is shown in Figure 2. Then it is cut, sanded, polished, etched and observed in a scanning electron microscope (SEM), and the average grain size is measured by the linear intercept method [32,33]. The experimental equipment used is GLEEBLE-1500D (Tsinghua University: Thermal-force Simulation Open Laboratory, Beijing, China). 

## 3. Application of an 33Cr23Ni8Mn3N Artificial Neural Network

**Figure 3:**
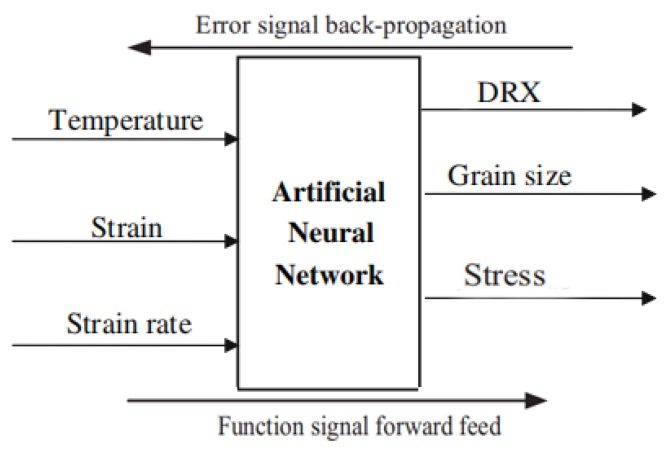
Schematic diagram of the artificial neural network (ANN) operation.

The ANN type used to establish the correlation model of the 33Cr23Ni8Mn3N is the BP-ANN. At present, the BP algorithm is one of the most used learning algorithms for contemporary researchers, and its application range is very wide. The advantage of the BP learning algorithm is that it has a strong learning ability and can continuously adjust weight through back-propagated information to achieve the minimum target error. Another advantage is the ability to describe complex multiple conditional couplings well [31]. Because of the above advantages, BP-ANN is very suitable for solving the target parameter values in this multi-conditional coupling. The ANN operation flow chart is shown in Figure 3. In the training process, when the training result is not ideal, the error information is returned. According to the error information of the back propagation, the weight is adjusted and training is performed again. This cycles back and forth until the training result reaches the set goal. The ANN is divided into three parts (input layer, hidden layer, and output layer). The number of neurons in the hidden layer can be adjusted to obtain the optimal training result. The number of neurons in this hidden layer is selected from 0–20. The final structure of the ANN model is determined by continuously training the ANN to different structures and comparing the error of the number of different neurons. When entering training data, the training data needs to be normalized first. The large deviation of the data values under different units will result in an increase in the deviation of the training results. The goal is to reduce the training convergence speed and accuracy problems caused by the measured data in different units by normalizing all training data (T, ε, $\dot{\varepsilon }$) to a dimensionless unit. The normalization function used in this training is the mapminmax function. After normalization, the value range of each input parameter is all [−1,1]. The mapminmax function expression is as follows:

(1) $$Y=\frac{2(X-X_{\min})}{X_{\max}-X_{\min}}-1$$

where X is the original vector value, X_min_ and X_max_ are the minimum and maximum values corresponding to X, respectively, and Y is the vector value normalized by the X vector.

### 3.1. ANN DRX Model

#### 3.1.1. ANN DRX Model and Accuracy Verification

Studies have shown that when the hidden layer is 1, it is enough to describe various model relationships well [34]. Therefore, the single hidden layer ANN structure is selected in this paper. The software used is Matlab2018a and the neural network type is BP-ANN. Selecting different functions for training observations is then necessary to determine the functions that are suitable for modeling. Finally, the parameters of the ANN model are determined, as shown in Table 1. The number of neurons in the hidden layer is determined by experience, and trial and error. The training effect is reasonably evaluated by introducing a mean square error (MSE) parameter. The equation is as follows:

(2) $$\mathrm{MSE}=\frac{1}{N}\sum_{i=1}^{N}(E_{i}-P_{i}{)}^{2}$$

where E is the experimental value of the X_drx_, P is the X_drx_ predicted by the ANN model, and N is the number of data points.

**Figure 4:**
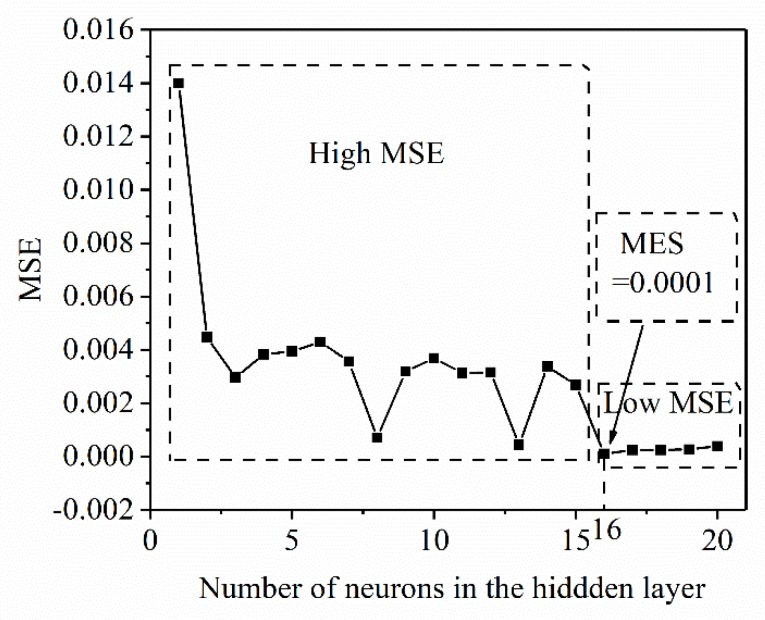
Effect of number of hidden layer neurons on prediction accuracy of the dynamic recrystallization (DRX) model.

**Figure 5:**
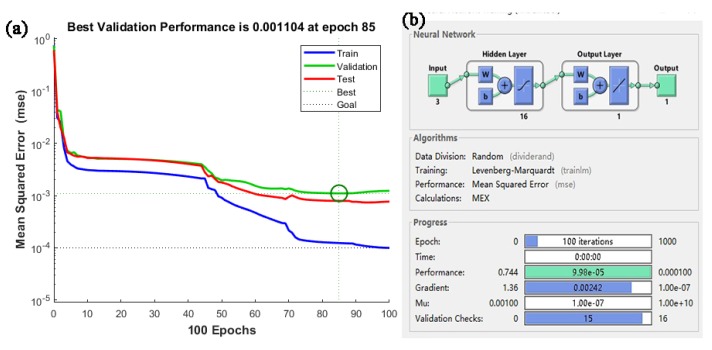
(**a**) Optimal ANN model training curve (**b**) and optimal ANN model structure and training parameters.

The relationship between the training error and the number of neurons (the number of neurons between 1 and 20) is shown in Figure 4. From the figure, it can be seen that the MSE of the prediction result shows the following law: in the high MSE region, the initial error decreases rapidly, and then a period of volatility (high MSE region) appears, and finally stabilizes (low MSE region). When the number of neurons is greater than 15, the prediction accuracy of the ANN is the highest. When the number of neurons is 16, the training error reaches the target of only 10^−4^. Finally, a single hidden layer neural network structure (3 × 16 × 1) is adopted, which has high prediction accuracy for the X_drx_ of 33Cr23Ni8Mn3N. Figure 5 shows the training parameters and training curves of the optimal ANN model. The set training target is 10^−4^. After 100 iterations, the system converges quickly and the training error reaches the set value. From Figure 5, it can be seen that the ANN model established by the above selected correlation parameters has the advantages of fast convergence speed and high precision.

After the X_drx_ prediction ANN model based on 33Cr23Ni8Mn3N heat-resistant steel is successfully established, X_drx_ is predicted by the ANN model. The prediction results are quantified using statistical parameters to evaluate the prediction accuracy of the ANN model. Three statistical parameters are mainly introduced: correlation coefficient (R), average root mean square error (e_RMSE_), and scattering index (Is). These equations are as follows:

(3) $$R=\frac{\sum_{i=1}^{n}\left(E_{i}-\bar{E})(P_{i}-\bar{P}\right)}{\sqrt{{\sum_{i=1}^{n}\left(E_{i}-\bar{E}\right)}^{2}{\left(P_{i}-\bar{P}\right)}^{2}}}$$

(4) $$e_{\mathrm{RMSE}}={\left[\frac{1}{N}\sum_{i=1}^{N}{\left(E_{i}-P_{i}\right)}^{2}\right]}^{1/2}$$

(5) $$I_{s}=\frac{e_{\mathrm{RMSE}}}{\bar{E}}$$

where $\bar{E}$, $\bar{P}$ is the average of E and P.

**Figure 6:**
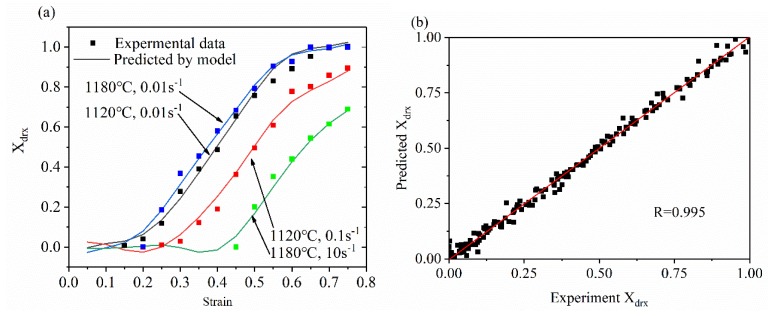
(**a**) Partial X_drx_ predicted values and experimental values (**b**) and X_drx_ predicted values and experimental values correlation.

When the X_drx_ is predicted by the ANN model (3 × 16 × 1), there is a problem in the prediction process in that there is a case where the prediction result for X_drx_ is slightly smaller than 0 and slightly larger than 1. The model is improved for this case, and the setting condition is that when the predicted value is slightly smaller than 0, the determination is 0, and when the predicted value is slightly larger than 1, the determination is 1. The prediction results are shown in Figure 6. It can be seen that the BP-ANN model can accurately predict the X_drx_ in the thermal deformation of 33Cr23Ni8Mn3N heat-resistant steel. This ANN model can provide some reference for X_drx_ control in 33Cr23Ni8Mn3N forming. For the prediction results, an evaluation based on statistical parameters was performed, with R = 0.995, e_RMSE_ = 0.022, and I_s_ = 0.054. e_RMSE_ is a parameter based on relative error calculation and is an unbiased statistic that measures the predictive power of the model. The e_RMSE_ is only 0.022, I_s_ is only 0.054, and R is as high as 0.995, indicating that the model has a strong prediction ability and high precision for the 33Cr23Ni8Mn3N thermal compression process.

#### 3.1.2. ANN DRX Grain Size Model Establishment

**Figure 7:**
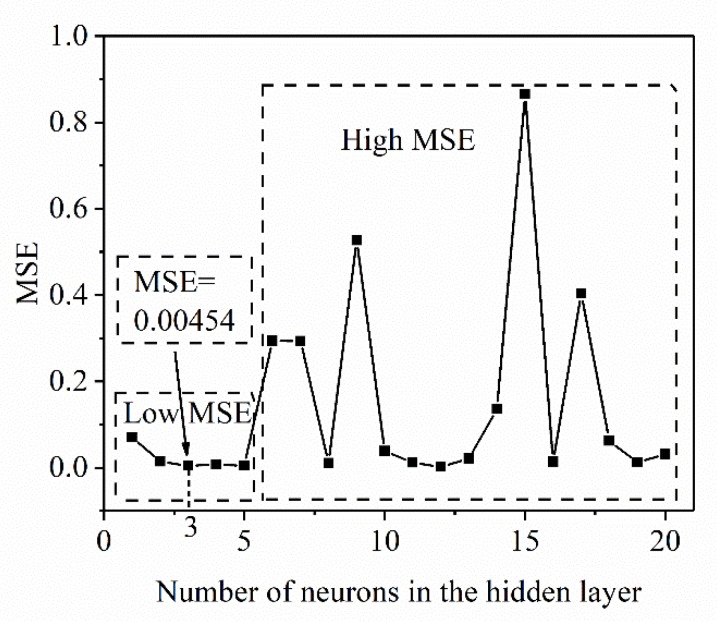
Effect of the number of hidden layer neurons on the prediction accuracy of the DRX grain size model.

**Figure 8:**
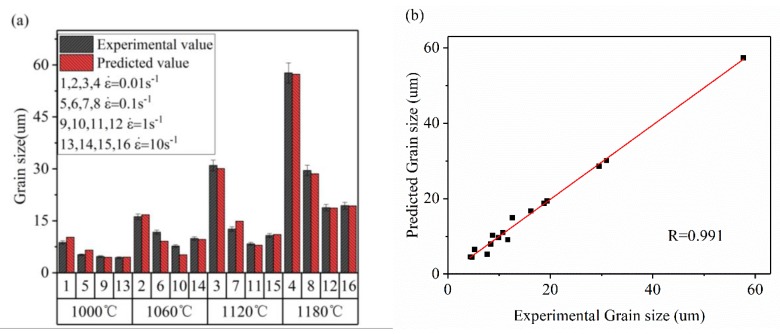
(**a**) DRX grain size prediction results (**b**) and correlation coefficient (R) of the DRX grain size prediction results.

For the establishment of the DRX grain size model, the same training parameters are used as shown in Table 1. For the accuracy of the ANN with different numbers of hidden layers, the results are shown in Figure 7. The training curve of the optimal ANN is substantially the same as that seen in Figure 5a and will not be described here. It can be seen that in the low MSE region, the training error fluctuation is small and low. So, the number of hidden layers is determined to be 3 (the best precision in the low MSE region). The DRX grain size was predicted by the trained ANN model (2 × 3 × 1), and the results are shown in Figure 8. As can be seen from Figure 8a, the experimental data is very close to the predicted data. Figure 8b shows the R between the experimental and predicted results under the same experimental conditions by regression analysis. R is as high as 0.991, which indicates that the ANN model has good precision for DRX grain size prediction and can provide theoretical guidance for DRX grain size during thermoforming.

#### 3.1.3. DRX Sensitivity Analysis

In order to analyze the degree of influence of deformation conditions on DRX, the SA was introduced to quantify it. Based on the analysis results, for models with many input parameters, the conditions of small importance can be removed to optimize the model structure [35]. There are many methods (Garson/Morris/Sobol) to quantify the importance of input parameters to results [20]. However, the current SA method proposed by Garson is the most recognized [24,36]. Therefore, this study used the Garson method to quantify the importance of input variables. The Garson method quantitatively determines the primary-secondary relationship of the input variables by connecting the weights of the trained ANN. This method is closely related to ANN and is suitable for this study with high reliability. The Garson method is as follows:

(6) $$S_{\mathrm{ik}}=\frac{\sum_{j=1}^{L}\left(|w_{\mathrm{ij}}v_{\mathrm{jk}}|/\sum_{r=1}^{N}|w_{\mathrm{rj}}|\right)}{\sum_{i=1}^{N}\sum_{j=1}^{L}\left(|w_{\mathrm{ij}}v_{\mathrm{jk}}|/\sum_{r=1}^{N}|w_{\mathrm{rj}}|\right)}$$

where $S_{\mathrm{ik}}$ is the relative importance of the input variables, $w_{\mathrm{ij}},v_{\mathrm{jk}}$ are the input layer—hidden layer, hidden layer—output layer connection weight, respectively, and i = 1, 2, ..., N; k = 1, 2, ... , L (N, M are the number of input variables and output variables, respectively).

**Figure 9:**
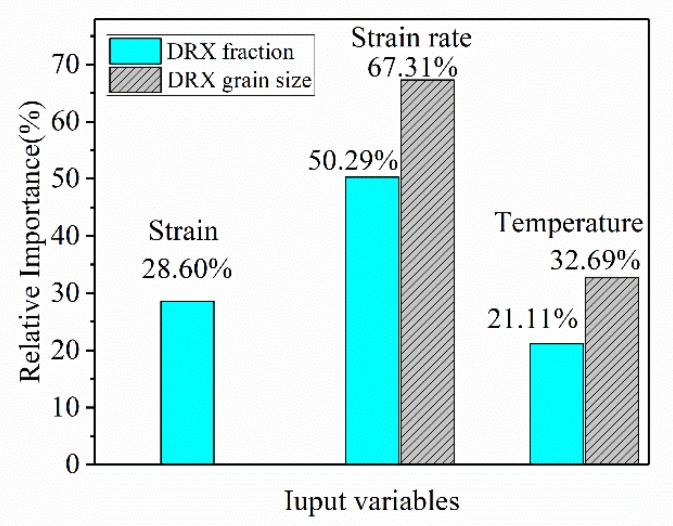
The importance of each variable in the DRX process.

The importance of each input variable for DRX is shown in Figure 9. The figure shows that the importance of DRX from high to low is $\dot{\varepsilon },\varepsilon ,T$. The most important parameter is $\dot{\varepsilon },$, which has a specific gravity of 50.29%. The other two variables have a greater impact on DRX, with importance values of 28.60% and 21.11%, respectively. The results show that for controlling the X_drx_ of the 33Cr23Ni8Mn3N heat-resistant alloy steel, it is preferred to control deformation condition $\dot{\varepsilon }$ with the best effect. For the SA of DRX grain size, it can be seen that $\dot{\varepsilon }$ has a very high influence on the DRX grain size, and its specific gravity is as high as 67.31%. Secondly, the importance of T is 32.69%, and it is not also negligible. Particularly when the T exceeds 1180 °C, the grain size rises sharply, so this condition should be paid attention to during thermoforming. The effect law of strain rate on DRX behavior is that as the $\dot{\varepsilon }$ increases, Xdrx decreases and the grain size decreases. The main reason is that DRX nucleation and growth take time. At high $\dot{\varepsilon }$, the deformation time is very short, the atomic diffusion time is short. The migration of grain boundary is realized by atomic diffusion, and the deformation process is completed before the newly generated fine equiaxial crystal has time to grow up. Therefore, the DRX is insufficient and the grain size is small. At low $\dot{\varepsilon }$, the deformation time is longer, the atoms have sufficient time to diffuse, and the recrystallized grains have sufficient time to nucleate and grow, so DRX is more sufficient and the grain size is larger at lower $\dot{\varepsilon }$. 

### 3.2. ANN Processing Map

This paper uses the most widely used dynamic material modeling (DMM) to establish the ANN processing map [37]. Based on theoretical DMM, input energy (P) can be divided into the dissipation amount (G) and the dissipation coordination (J). The expressions of the two are:

(7) $$P=G+J=\int_{0}^{\dot{\varepsilon }}\sigma d\dot{\varepsilon }+\int_{0}^{\sigma }\dot{\varepsilon }d\sigma$$

The expression of the strain rate sensitivity index (m) value of a metal material during thermal processing is:

(8) $$m={\frac{\partial (\mathrm{lg}\sigma )}{\partial (\mathrm{lg}\dot{\varepsilon })}|}_{\varepsilon ,T}$$

The power dissipation efficiency factor (η) represents the energy consumed by the evolution of the microstructure, and the relationship is:

(9) $$\eta =\frac{2m}{m+1}$$

The instability criterion used in this paper is the Prasad instability criterion [38]:

(10) $$\xi (\dot{\varepsilon })=\frac{\partial [\ln(\frac{m}{m+1})]}{\partial (\ln\dot{\varepsilon })}+m<0$$

**Figure 10:**
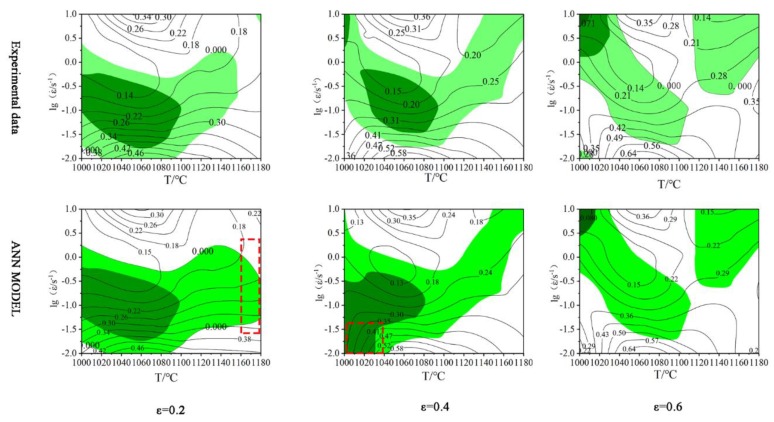
Comparison of processing map: the shaded areas are unstable areas, and the probability of instability is proportional to the color depth.

**Figure 11:**
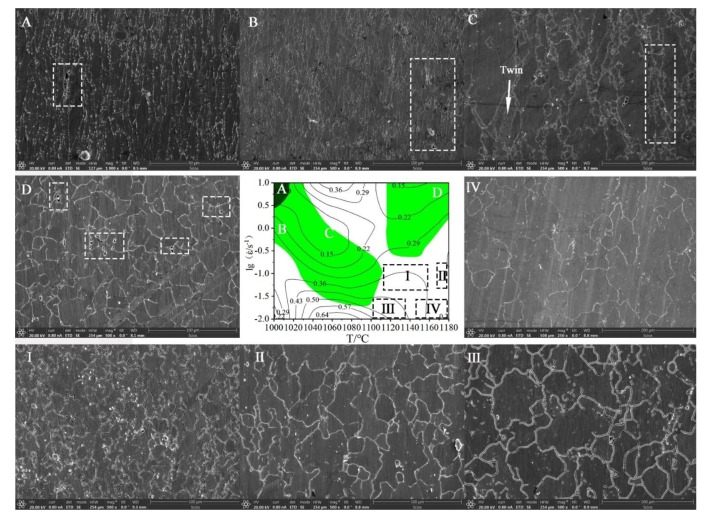
ANN processing map microstructure of different regions: (**A**–**D**,**I**–**IV**) are the microstructures under the corresponding processing parameters.

In the previous study, a BP-ANN model based on 33Cr23Ni8Mn3N heat-resistant steel has been established [39]. The R between the experimental value and the predicted value and average absolute relative error (AARE) are 0.999 and 0.697%, respectively. This paper establishes the ANN processing map using the predicted data of this model. In the processing map, the shaded area is the unstable area. The unstable area is prone to material instability, which leads to a sharp decrease in material properties or even scrap. The darker the shade, the higher the probability of instability. The ANN processing map is compared with the processing map based on the experimental data and is shown in Figure 10. It can be seen from the comparison graph of the two that the peak η appears below and the peak value is the same (46%, 58%, and 64%). Comparing the experimentally based processing maps, the unstable regions (shaded portions) and the peak η regions of the ANN processing maps are basically the same. The unstable region of the ANN processing map is slightly larger (the excess is shown in the shaded area inside the red dashed box in Figure 10), and the information of the ANN processing map is more detailed and accurate, so that the optimal process parameter interval can be determined more accurately and easily. Figure 11 shows the microstructures of different regions of the ANN processing map (ε = 0.6), and the microstructures of different unstable and safe regions are verified. Among them, A, B, C, D, I, II, III, and IV correspond to the corresponding processing parameter areas in the processing map. The A region shows a large probability of instability, and cracks and voids affecting the material properties are observed in the microstructure (Figure 11A). The B and D regions are unstable, and voids and flow localization are observed in the microstructure, which causes the material properties to decrease. Significant twinning and mixed grains appear in the unstable region C. A "necklace" structure appears, and the original grains are mixed with the DRX grains. The safe regions I, II, III, IV have η values of more than 0.3 and are not covered by the unstable region, making them ideal thermoforming regions. However, the microstructures of the safe regions II, III, IV have grown up, and there are many precipitates which are very harmful to the material. The microstructure of the safe region I is uniform, fine, and has a few precipitates, and the precipitates are not meshed, which is the best choice for the optimal process parameters. Therefore, the same conclusion is obtained for the ANN processing map. The optimal process parameters are T = 1120–1160 °C, $\dot{\varepsilon }$ = 0.03–0.1 s^−1^. The prediction accuracy of the ANN model for the stress (σ) of 33Cr23Ni8Mn3N heat-resistant alloy steel is as high as 0.999 [39]. The processing map under different experiment conditions can be established by predicting data. In this way, the ANN processing map has the advantages of economy and high efficiency.

### 3.3. Application of ANN Constitutive Model in Finite Element Simulation

In this paper, the feasibility of an ANN model in finite element simulation modeling is be studied, and the ANN model is embedded into the finite element simulation software DEFORM-3D (V6.1, Science Forming Technology Corporation, Clumbus, OH, USA). The feasibility and reliability of the ANN constitutive model in finite element simulation are verified by simulated thermal compression experiments. In DEFORM-3D, two sets of experimental simulations (1120 °C, 1 s^−1^ and 1180 °C, 1 s^−1^) were performed. The experimental parameters are as follows: the mold is set to rigid and the workpiece is set to plastic. The heat exchange index with the environment is 0.02 N/sec/mm/°C, the heat transfer coefficient between the workpiece and the mold is 11 N/sec/mm/°C, the mold T is 20 °C, and the number of meshes for the workpiece is 60,000. This is because the friction of the end face of the sample is the main factor affecting the accuracy of the test. In the actual thermal compression process, graphite sheets need to be placed between the workpiece and the mold to reduce its influence on the accuracy of the results. In the simulation, the shear friction coefficient of the workpiece and the mold is 0.2 to simulate actual working conditions.

**Figure 12:**
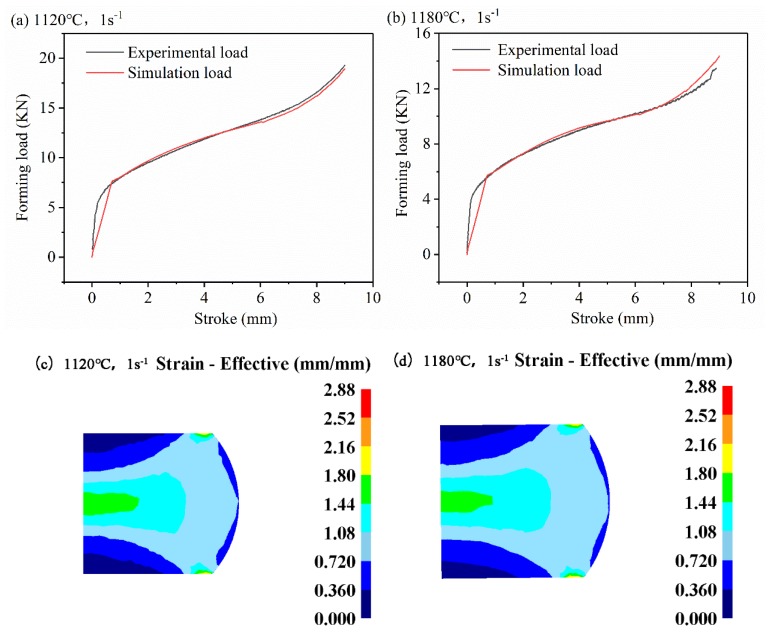
Numerical simulation results: Mold load at (**a**) 1120 °C, 1s^−1^ (**b**) and 1180 °C, 1s^−1^ strain distribution at (**c**) 1120 °C, 1 s^−1^ (**d**) and 1180 °C, 1 s^−1^.

The simulation results are shown in Figure 12. Figure 12a,b are comparisons of mold loads under experimental conditions (1120 °C, 1 s^−1^ and 1180 °C, 1 s^−1^). The experimental load is collected by the information acquisition system of the GLEEBLE-1500D equipment (DATA SCIENCES INTERNATIONAL, INC., MI, USA) under the test conditions. From the figure, it can be seen that the simulated load-stroke curve is very consistent with the actual experiment data, and the variation law is similar. At the beginning of the deformation (0–1 mm), the load increases rapidly, and in the next deformation stage (1–7 mm), the steady increase state is maintained, and in the final stage (7–9 mm) load increases again rapidly. Figure 12c,d show the ε distribution on the center section of the workpiece under experimental conditions (1120 °C, 1 s^−1^ and 1180 °C, 1 s^−1^). The deformation distribution law of the two is generally the same: the deformation of the center of the workpiece is the largest (called the large deformation zone), the deformation of the upper and lower positions of the workpiece is the smallest (called the free zone), and the deformation of the edge of the workpiece is between the two (called the adhesive area). The ε distribution law obtained by the ANN constitutive model simulation is consistent with the experimental law obtained by the ideal isothermal compression test, which is consistent with the thermal compression ε distribution law obtained by previous scholars [40,41]. This shows that it is feasible to carry out a finite element simulation of a 33Cr23Ni8Mn3N heat-resistant alloy by using an ANN model. 

**Figure 13:**
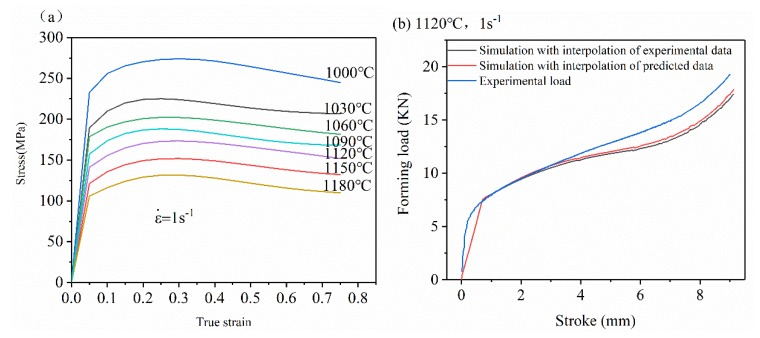
(**a**) ANN model prediction data (**b**) and different interpolation conditions by mold load comparison.

When using the interpolation method to simulate the hot deformation process, the simulation accuracy depends on the number of experimental data of the interpolation input, and the accuracy increases as the number increases. However, due to economic factors, equipment raw materials, time constraints, and other factors, it is not feasible to conduct a large number of experimental tests. It is neither realistic nor economical, and is not an economic benefit for enterprises. Therefore, limited experimental data is one of the most important reasons for restricting the accuracy of finite element simulation. Through the high prediction accuracy of the ANN model, the experimental data is predicted by a large amount, and the prediction data is the input into the finite element simulation software, thereby improving the precision loss due to a limited amount of data. In order to verify whether this theory is feasible for a 33Cr23Ni8Mn3N heat-resistant alloy steel, two sets of DEFORM finite element simulations are performed. First, the ANN model is used to predict different T data under $\dot{\varepsilon }$ is 1 s^−1^, and the result is shown in Figure 13a. The interpolation method is used for the simulated constitutive relation. The input data of the two groups is as follows: group 1 inputs experimental data of 1000, 1060, and 1180 °C, and group 2 inputs prediction data of 1000, 1030, 1060, 1090, 1150, and 1180 °C. The finite element simulation of isothermal compression experiments of 33Cr23Ni8Mn3N at 1120 °C, 1 s^−1^ was carried out. During the simulation, the system automatically inserted the data under deformation conditions. Figure 13b shows the comparison of mold load and experimental data for different interpolated data volumes to verify the prediction accuracy of different methods. The simulation of the group with more data is closer to the actual experimental situation. As the amount of interpolated data increases, it becomes more consistent with the actual experiment. This indicates that the ANN model is of great significance to the improvement of simulation accuracy when the interpolation method is used to conduct finite element simulation research on the thermal deformation behavior of 33Cr23Ni8Mn3N heat-resistant alloy steel.

## 4. Conclusions

(1) Based on BP-ANN, the prediction accuracy of different neurons in the hidden layer has been evaluated. When the number of neurons was 16–20, the prediction accuracy was high and the error fluctuation was small. When the number of neurons was 16, the highest accuracy was achieved. The relevant values were R = 0.995, e_RMSE_ = 0.022, and I_s_ = 0.054, which indicates that the X_drx_ model established by ANN technology in this study is suitable for describing the relationship between thermal deformation parameters and X_drx_ during the thermoforming process of 33Cr23Ni8Mn3N heat-resistant steel.
(2) The DRX grain size model was established by an ANN, and its prediction value accuracy was at high R = 0.991. For the special case where the DRX grain size rose sharply at a T value of 1180 °C, accurate predictions can also be made.
(3) The SA of each deformation parameter in the 33Cr23Ni8Mn3N DRX process showed that the effect of $\dot{\varepsilon }$ on DRX was the most important. For the control of microstructure during processing, the $\dot{\varepsilon }$ was preferentially controlled, and the effect was the best and the sensitivity was the highest.
(4) The ANN processing map reflection information was basically consistent with the processing map based on experiment data. The unstable region was slightly larger, but did not affect the determination of the optimal process parameters, and the ANN processing map was more detailed and made it easier to determine the optimal process parameters. In the optimal process parameter interval determined by the ANN processing map, the microstructure had the advantages of being uniform, fine, and having less precipitates. When the process parameters were 1120 °C, 0.01 s^−1^ and 1180 °C, 0.1 s^−1^, there were many precipitates, and the precipitates were connected into reticulation. When selecting process parameters, this should be avoided.
(5) The ANN was applied to the modeling and simulation of the 33Cr23Ni8Mn3N alloy. Through the means of simulation verification and experimental comparison, the feasibility of an ANN in 33Cr23Ni8Mn3N simulation was proven, and the application potential wa high and can be applied widely. With the ability to accurately model through limited experimental data, the ANN model has the advantages of economy and efficiency. The ANN model is of great significance to the improvement of simulation accuracy when the interpolation method is used to conduct finite element simulation research on the thermal deformation behavior of 33Cr23Ni8Mn3N heat-resistant alloy steel.

## Figures and Tables

**Table 1:** Artificial neural network (ANN) training parameters.

PARAMETER	CONTENT
Neural network type	BACK PROPAGATION
Adaption learning function	LEARNGM
Training function	TRAINLM
Transfer Function (input and hidden layer)	TANSIG
Activation Function (hidden layer to output layer)	PURELIN
Performance function Training epoch Goal	MSE 1000 10^−4^
